# Lack of cytomegalovirus detection in human glioma

**DOI:** 10.1186/s12985-017-0885-3

**Published:** 2017-11-07

**Authors:** Araceli Garcia-Martinez, Cristina Alenda, Esperanza Irles, Enrique Ochoa, Teresa Quintanar, Alvaro Rodriguez-Lescure, Jose L. Soto, Victor M. Barbera

**Affiliations:** 1 Molecular Genetics Laboratory, Elche University General Hospital, Alicante Institute for Health and Biomedical Research (ISABIAL - FISABIO Foundation), Alicante, Spain; 2 Department of Pathology, Alicante University General Hospital, Alicante Institute for Health and Biomedical Research (ISABIAL - FISABIO Foundation), Alicante, Spain; 3 Molecular Biopathology Unit, Provincial Hospital of Castellón, Castellón, Spain; 4 Medical Oncology Department, Elche University General Hospital, Elche, Spain

**Keywords:** Glioma, Human cytomegalovirus, Gliomagenesis, Detection

## Abstract

Gliomas are the most common brain tumors and include a variety of histologic types and grades of malignancy. They arise from glial cells and represent approximately 70% of the primary brain tumors. According to the criteria of the World Health Organization (WHO), the majority of gliomas can be classified into four grades of malignancy (I-IV). Virus infection, especially by DNA viruses and retroviruses, which may cause insertion of viral DNA sequences into the host genome, often triggers the host defense mechanisms. Particularly, the DNA methylation machinery can be activated to cause the methylation of foreign movable viral sequences and, therefore, silence viral gene expression. Several studies have shown the presence of Human Cytomegalovirus (HCMV) in glioblastoma, suggesting that the virus may participate in tumor pathogenesis. But this relationship is controversial because many other studies did not detect HCMV in these tumors. This study aims to detect the presence of HCMV in several samples of human glioma (94 formalin-fixed, paraffin-embedded samples and 28 snap-frozen samples) by different sensitive techniques. We have been unable to detect HCMV DNA and proteins in glioma samples. Therefore, arguments used so far to conclude that HCMV is an oncomodulator virus in gliomas must be, in our view, seriously reconsidered.

**Electronic supplementary material:**

The online version of this article (10.1186/s12985-017-0885-3) contains supplementary material, which is available to authorized users.

## Background

Gliomas constitute a heterogeneus group of malignant neoplasms of the central nervous system which are derived from glial cells and represent the most frequent form of primary brain tumors, over 70% of cases [1, 2]. Gliomas are classified into four malignant grades attending to their morphological and histological features. Glioblastoma multiforme, the most malignant type of glioma (WHO grade IV), is highly aggressive and invasive, with a mean survival rate of 14–15 months after diagnosis.

In gliomas, the etiological factors still remain elusive. Yet, although large epidemiological studies have failed to identify new clear causative factors, in recent years cytomegalovirus infections have focused the scientific community attention. However, their etiological role in the genesis of gliomas is currently controversial.

HCMV is a double-stranded DNA virus of the Herpesviridae family with a genome of ca. 230 kb and containing ca. 200 genes coding for viral proteins and at least 14 microRNAs [3, 4]. HCMV is extremely prevalent, with infections rates between 50 and 90% of adult population [5]. Since the finding of HCMV in brain tumors in 2002 by Cobbs et al. [6], controversy about the presence of HCMV in glioma genomes has increased in the literature. Viral DNA, RNA and proteins were HCMV-positive in the majority of tumor cells in human glioblastoma, including both anaplastic and low-grade gliomas [6, 7]. However, these studies have used distinct and non-uniform methodological approaches, and the same accounts for the target molecules used for virus detection. This complete lack of standarization thus constitutes in our view a crucial problem. Additionally, the controversy on the implication of HCMV in gliomas has been fed with data pointing out that anti-viral drugs could improve the survival outcome to this disease.

### Procedure and results

With the aim of shedding light on such uncertainty, we set to analyze in this study a large series of primary gliomas in order to detect the presence of HCMV by a diagnostic, validated real-time PCR method. With this purpose, we analyzed a panel of 68 formalin-fixed, paraffin-embedded samples and 28 snap-frozen samples from patients with astrocytoma obtained from the biobank of the Hospital General Universitario de Elche, together with 26 formalin-fixed, paraffin-embedded samples from patients with astrocytoma from the biobank of the Hospital General Universitario de Alicante (total, 122 samples; Table 1). Additionally, a formalin-fixed, paraffin-embedded tissue sample of lung infected with HCMV was used as a positive control.

**Table 1:** Patient characteristics

Variable	Number	Percent
Gender		
Male	72	59
Female	50	41
Age (years)		
Mean	54	
Range	4-81	
Tumor grade		
II	16	13.1
III	19	15.6
IV	87	71.3

Genomic DNA was isolated from tumor and control tissues using the high performance QIAamp DNA Investigator forensic kit. HCMV DNA detection in glioma samples was performed with 5 uL of isolated genomic DNA per RT-PCR reaction in an Applied Biosystems 7500 Real-Time PCR System using a very sensitive diagnostic validated test, RealStar CMV PCR kit 1.0 (Altona Diagnostics GmbH, Hamburg, Germany). This assay includes an internal control (heterologous amplification system) and in each reaction we included the four standardized concentrations of HCMV specific DNA (10^1^-10^4^ genome copies) supplied with the kit. As a positive control we used genomic DNA from formalin-fixed, paraffin-embedded lung tissue of an infected donor. For HCMV DNA analysis, a four-point standard curve (Pearson’s correlation coefficient, >0.99) was used to interpolate the HCMV viral load from 10 to 10,000 copies (Additional file 1). Surprisingly, 119 samples of the whole set of gliomas analyzed showed undetectable levels of HCMV DNA, and only three cases showed a low number (<10) of viral copies (Table 2).

**Table 2:** Results of the different techniques used

Technique	Number of samples	HCMV-positive	HCMV-negative
RT-qPCR	122	3	119
Nested PCR	30	0	30
IHC	110	0	110

A nested PCR approach was used in a selected subset of samples. Genomic DNAs from two formalin-fixed, paraffin-embedded and 28 snap-frozen samples were processed following a nested-PCR scheme [8] (Additional file 1). Using this technique, no sample was found positive for HCMV DNA (Table 2). Yet, we believe that this methodology is not completely reliable, since the number of PCR amplification cycles is extremely high (up to 90 cycles), which represents an important risk of DNA cross-contamination.

Immunohistochemical (IHC) evaluation was performed on sections of tissue microarrays from 110 samples (25 snap-frozen samples and 85 formalin-fixed, paraffin-embedded samples). We used an optimized antibody (Anti-cytomegalovirus antibody cocktail CCH2/DDG9 Dako), corresponding to HCMV-encoded p52/p76 kDa early DNA-binding protein and early protein [9]) (Additional file 1). No sample was found positive by IHC using this approach (Table 2).

## Discussion

Several groups [7, 8, 10–12] have been able to detect HCMV DNA and proteins in glioma samples in the same proportion as that obtained by Cobbs et al. 2002, i.e. of 90-100%. Other groups have reported virus detection in glioma samples, but in a lower proportion (12-70%) [13–16]. Finally, some groups [9, 17–21] have been unable to detect neither HCMV DNA or proteins. This discrepancy is likely attributable to methodological differences among these studies [22]. In this context, a recent published paper supports our results [23]. They used 6 highly sensitive assays with three orthogonal technologies (real-time PCR, IHC and CISH) on multiple specimens and specimen types. No evidence for CMV in glioblastoma tissues was found.

The HCMV infection level in glioma samples in most studies is usually very low, and thus more sensitive methods may be required to detect persistent HCMV infection in tumor cells [24]. Also, optimized antigen retrieval and a high antibody concentration are thought to be the key factors required for HCMV detection by IHC methods [9].

Since at present HCMV is not considered to be an oncogenic virus, the term oncomodulation has been proposed to designate the ability of HCMV to modify tumor cell biology [25–28]. There are currently several hypotheses on how HCMV enters the human brain, but none of them implies that the virus plays a role in gliomagenesis. Thus, it is not clearly defined still whether HCMV infection is an epiphenomenon or is actually causative of this type of tumors [26]. Given that the infection rate is very low and no studies have yet attempted HCMV detection by means of transcriptomic or genomic DNA analyses using next-generation sequencing, it must be evaluated whether HCMV can by itself elicit oncogenic and immunosuppressive processes, and consequently whether this virus is actually a relevant therapeutic target in glioblastomas [22]. Also, despite there are numerous ongoing clinical trials, no evidence has been yet obtained on the biological efficacy of anti-viral agents on gliomas. It must also be taken into account that anti-viral treatments may also act in other ways independent of HCMV targeting, for instance by synergizing with chemotherapy and/or radiation therapies [29, 30].

## Conclusions

In conclusion, there is currently a great controversy on the relationship between HCMV and gliomagenesis. A multicenter study is necessary in which a standardized methodology is used to unequivocally determine whether this virus is actually present in glioma samples. We believe that at present anti-viral treatments should not be used outside clinical trials in glioma patients. Our group has analyzed in this work a large number of samples using high specificity and sensitive methods, and as a result we have been unable to detect HCMV DNA and proteins in glioma samples. Therefore, arguments used so far to conclude that HCMV is an oncomodulator virus in gliomas must be in our view seriously reconsidered.

## Additional file

Additional file 1: Methodological details and proceduries. (DOCX 40 kb)

## Abbreviations

HCMV: Human cytomegalovirus
IHC: Immunohistochemical
WHO: World Health Organization
