# Supplementary material for: Lack of cytomegalovirus detection in human glioma
Source: Virol J. 2017 Nov 7;14:216. doi: 10.1186/s12985-017-0885-3 (PMC5678593; doi:10.1186/s12985-017-0885-3)
Supplement: Additional file 1: — Methodological details and proceduries. (DOCX 40 kb) [file 12985_2017_885_MOESM1_ESM.docx]

**Additional file 1**

**CMV detection with RealStar CMV PCR kit 1.0 (*Altona Diagnostics GmbM*)**

The RealStar® CMV PCR Kit 1.0 is an *in vitro* diagnostic test, based on real-time PCR technology, for the detection and quantification of Cytomegalovirus (CMV) specific DNA.

The RealStar® CMV PCR Kit 1.0 is an *in vitro* diagnostic test, based on real-time PCR technology, for the detection and quantification of CMV specific DNA. The assay includes a heterologous amplification system (Internal Control) to identify possible PCR inhibition and to confirm the integrity of the reagents of the kit. The test is based on real-time PCR technology, utilizing polymerase chain reaction (PCR) for the amplification of specific target sequences and target specific probes for the detection of the amplified DNA. The probes are labelled with fluorescent reporter and quencher dyes.

Probes specific for CMV DNA are labelled with the fluorophore FAM. The probe specific for the Internal Control (IC) is labelled with a fluorophore showing the same characteristics as Cy3. Using probes linked to distinguishable dyes enables the parallel detection of CMV specific DNA and the Internal Control in corresponding detector channels of the real-time PCR instrument.

The test consists of two processes in a single tube assay: PCR amplification of target DNA and Internal Control and simultaneous detection of PCR amplicons by fluorescent dye labelled probes.

The RealStar® CMV PCR Kit 1.0 consists of:

- Two Master reagents (Master A and Master B)
- Template Internal Control (IC)
- Four Quantification Standards (QS1 – QS4)
- PCR grade water

Master A and Master B reagents contain all components (buffer, enzymes, primers and probes) to allow PCR mediated amplification and target detection of CMV specific DNA and Internal Control in one reaction setup. The Quantification Standards (QS) contain standardized concentrations of CMV specific DNA. These Quantification Standards were calibrated against the 1st WHO International Standard for Human Cytomegalovirus for Nucleic Acid Amplification Techniques (NIBSC code: 09/162). The Quantification Standards can be used individually as positive controls, or together to generate a **standard curve**, which can be used to determine the concentration of CMV in the sample. The following concentrations are used:

| **Quantification Standards** | **Concentration [IU/μl]** |
| --- | --- |
| QS1 | 1.00E+04 |
| QS2 | 1.00E+03 |
| QS3 | 1.00E+02 |
| QS4 | 1.00E+01 |

Extracted DNA is the starting material for the RealStar® CMV PCR Kit 1.0. The quality of the extracted DNA has a profound impact on the performance of the entire test system. It has to be ensured that the system used for nucleic acid extraction is compatible with real-time PCR technology. All reagents and samples should be thawed completely, mixed (by pipetting or gentle vortexing) and centrifuged briefly before use. The RealStar® CMV PCR Kit 1.0 contains a heterologous Internal Control (IC).

Reaction mix

| \| Number of Reactions (rxns) \| \| --- \| | **1** |
| --- | --- | --- |
| **Máster A** | 2.5µL |
| **Máster B** | 5µL |
| **Internal control** | 0.5µL |
| Volume | 8µL |

qPCR conditions

| 2 min a 95ºC 1 cycle |
| --- |
| 5 sec a 95ºC |
| 30 sec a 60ºC  45 cycles |
| 10 sec a 72ºC |
| 30 sec a 40ºC 1 cycle |

Samples were analyzed in duplicate. A standard six-point curve (Pearson's correlation coefficient> 0.99) was used to interpolate the HCMV viral load from 10 to 10,000 copies per cell.

**Nested PCR**

The method was obtained from the article "Genetic Analysis of Cytomegalovirus in Malignant Gliomas" (Bornali Bhattacharjee, NicholascRenzette and Timothy F.Kowalik; Journal of Virology, June 2012, Volume 86 Number 12). External primers amplify a 1.3 kbp region of the "major immediate-early promoter ". Internal primers amplify a region inside the amplicon of 1.3kbp generated by the external primers:

CMVextern_forward: **5’-CCGAAATACGCGTTTTGAGAT-3’**

CMVextern_reverse: **5’-CCAAGCCAAAAACAGTATAGC-3’**

CMVintern_forward: **5’-GGCGGAGTTRTTACGACATTT-3’**

CMVintern_reverse: **5’-ATGCGGTTTTGGCAGTACAT-3’**

The primers reached a concentration of 100 ppm. They were resuspended and an aliquot was made at 20ppm (4μL of primer at 100ppmol + 16μL water). For the first test, two samples of gliomas (HGUE) from paraffin, a positive control (paraffin) and a negative control (water) were used:

- The volume of each sample was calculated to obtain 100ng of DNA.
- First PCR:

| **Reagents** | **Sample 1** | **Sample 2** | **+ control** | **- control** |
| --- | --- | --- | --- | --- |
| Amplitaq (µL) | 10 | 10 | 10 | 10 |
| Glicerol 50% (µL) | 3.2 | 3.2 | 3.2 | 3.2 |
| PrimerExt_F (20pmol) | 0.625 | 0.625 | 0.625 | 0.625 |
| PrimerExt_R (20pmol) | 0.625 | 0.625 | 0.625 | 0.625 |
| DNA (100ng) (µL) | 1.15 | 1.4 | 0.9 | 2 |
| H_2_O (µL) | 4.4 | 4.15 | 4.65 | 3.55 |
| Final volume (µL) | **20** | | | |

- Conditions of the first PCR:

50 cycles


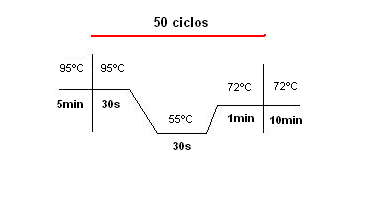


- Second PCR:

| **Regents** | **Sample 1** | **Sample 2** | **+ control** | **- control** |
| --- | --- | --- | --- | --- |
| Amplitaq (µL) | 10 | 10 | 10 | 10 |
| Glicerol 50% (µL) | 3.2 | 3.2 | 3.2 | 3.2 |
| PrimerInt_F (20pmol) | 0.625 | 0.625 | 0.625 | 0.625 |
| PrimerInt_R (20pmol) | 0.625 | 0.625 | 0.625 | 0.625 |
| DNA (First PCR product) (µL) | 4 | 4 | 4 | 4 |
| H_2_O (µL) | 1.55 | 1.55 | 1.55 | 1.55 |
| Final volume (µL) | **20** | | | |

- Conditions of the second PCR:

40 cycles


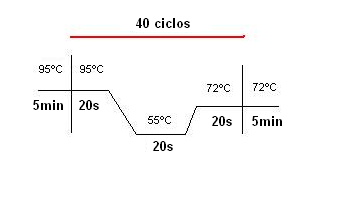


A second experiment was performed with 25 glioma samples from frozen HGUE tissue. The procedure was exactly the same as the one performed previously.

**Immunohistochemistry**

Immunohistochemical staining was performed using a Dako Omnis System (Dako, Agilent Technologies, Carpinteria, CA, USA), according to the manufacturer's instructions. Antigen retrieval was performed using EnVision FLEX Target Retrieval Solution, High pH (Dako, Agilent Technologies). Sections were incubated for 25 minutes with primary antibody against cytomegalovirus (clone CCH2/DDG9, Dako, Agilent Technologies). After chromogenic visualization, using EnVision FLEX/HRP (Dako, Agilent Technologies), slides were counterstained with hematoxylin.

***In situ* hibridation**

*In situ* hibridation was performed with Bond™ Ready-to-Use ISH CMV Probe (Leyca Biosystems). CMV Probe is intended to be used for the qualitative identification of human cytomegalovirus early gene RNA transcript, in formalin-fixed, paraffin-embedded tissue by in situ hybridization (ISH) using the automated BOND system (includes Leica BOND-MAX system and Leica BOND-III system) in combination with Anti-Fluorescein Antibody and Bond Polymer Refine Detection. Tissue and reagent controls were used.
